# Responses of Epibranchial Placodes to Disruptions of the FGF and BMP Signaling Pathways in Embryonic Mice

**DOI:** 10.3389/fcell.2021.712522

**Published:** 2021-09-13

**Authors:** Stefan Washausen, Wolfgang Knabe

**Affiliations:** Prosektur Anatomie, Westfälische Wilhelms-Universität Münster, Münster, Germany

**Keywords:** epibranchial placodes, neurogenesis, induction, pharyngeal pouches, signaling centers, fibroblast growth factors, bone morphogenetic proteins, mouse whole embryo culture

## Abstract

Placodes are ectodermal thickenings of the embryonic vertebrate head. Their descendants contribute to sensory organ development, but also give rise to sensory neurons of the cranial nerves. In mammals, the signaling pathways which regulate the morphogenesis and neurogenesis of epibranchial placodes, localized dorsocaudally to the pharyngeal clefts, are poorly understood. Therefore, we performed mouse whole embryo culture experiments to assess the impact of pan-fibroblast growth factor receptor (FGFR) inhibitors, anti-FGFR3 neutralizing antibodies or the pan-bone morphogenetic protein receptor (BMPR) inhibitor LDN193189 on epibranchial development. We demonstrate that each of the three paired epibranchial placodes is regulated by a unique combination of FGF and/or bone morphogenetic protein (BMP) signaling. Thus, neurogenesis depends on fibroblast growth factor (FGF) signals, albeit to different degrees, in all epibranchial placodes (EP), whereas only EP1 and EP3 significantly rely on neurogenic BMP signals. Furthermore, individual epibranchial placodes vary in the extent to which FGF and/or BMP signals (1) have access to certain receptor subtypes, (2) affect the production of Neurogenin (Ngn)2^+^ and/or Ngn1^+^ neuroblasts, and (3) regulate either neurogenesis alone or together with structural maintenance. In EP2 and EP3, all FGF-dependent production of Ngn2^+^ neuroblasts is mediated via FGFR3 whereas, in EP1, it depends on FGFR1 and FGFR3. Differently, production of FGF-dependent Ngn1^+^ neuroblasts almost completely depends on FGFR3 in EP1 and EP2, but not in EP3. Finally, FGF signals turned out to be responsible for the maintenance of both placodal thickening and neurogenesis in all epibranchial placodes, whereas administration of the pan-BMPR inhibitor, apart from its negative neurogenic effects in EP1 and EP3, causes only decreases in the thickness of EP3. Experimentally applied inhibitors most probably not only blocked receptors in the epibranchial placodes, but also endodermal receptors in the pharyngeal pouches, which act as epibranchial signaling centers. While high doses of pan-FGFR inhibitors impaired the development of all pharyngeal pouches, high doses of the pan-BMPR inhibitor negatively affected only the pharyngeal pouches 3 and 4. In combination with partly concordant, partly divergent findings in other vertebrate classes our observations open up new approaches for research into the complex regulation of neurogenic placode development.

## Introduction

Ectodermal placodes are essential for the formation of sensory organs and parts of the peripheral nervous system of the vertebrate head (Baker and Bronner-Fraser, 2001; Schlosser, 2006; Park and Saint-Jeannet, 2010). They are excellent objects for basic research in developmental biology. This applies first of all to the question to which extent genetic patterning mechanisms, migratory activities and programmed cell death help to transform the “panplacodal primordium” into individual patch-like thickened placodes (Washausen et al., 2005; Schlosser, 2010; Washausen and Knabe, 2013, 2019; Breau and Schneider-Maunoury, 2014; Thiery et al., 2020). Unanswered questions also concern the developmental potential of the panplacodal primordium in different vertebrate classes. For example, in normal developing mice, posterior parts of the primordium (“posterior placodal area”; Schlosser and Ahrens, 2004; Washausen and Knabe, 2017) exclusively produce otic and epibranchial placodes, the latter ones providing gustatory and other viscerosensory neurons for ganglia associated with the facial, glossopharyngeal and vagal nerves. However, experimental suppression of physiologically occurring apoptosis in mouse whole embryo cultures results in the additional generation of lateral line placodes (Washausen and Knabe, 2018). Finally, numerous gaps exist regarding our knowledge about the signaling centers and signaling cascades which are involved in placode formation.

This work focuses on the signaling centers and pathways which support the development of epibranchial placodes in mouse embryos. According to current knowledge, demarcation of the panplacodal primordium from the neural plate, neural crest and surface ectoderm requires fibroblast growth factor (FGF), bone morphogenetic protein (BMP), and Wingless/Int-1 (Wnt) signals. They are provided by the epiblast, by the cephalic mesoderm and by the future neural plate, among others (Streit, 2018). Subsequently, FGF signals from the cephalic mesoderm help to delineate the posterior placodal area. Cell fate decisions that follow pave the way for the assembly of progenitor cells into epibranchial and otic placodes, respectively. They are supported by Wnt signals provided by the hindbrain (Ladher et al., 2010; Chen and Streit, 2013). Regarding the signaling pathways which regulate the morphogenesis and neurogenesis of epibranchial placodes, most extensive findings are available for zebrafish. Specifically, this involves BMP and FGF signals, which are released from the pharyngeal pouches (Holzschuh et al., 2005; Nechiporuk et al., 2005, 2007). Similarly, in chicken embryos, contributions of mesodermal FGF3/FGF19 and pharyngeal BMP signals have been documented (Begbie et al., 1999; Freter et al., 2008; Kriebitz et al., 2009). All that is known about mouse embryos in this context is that neurogenesis of the first epibranchial placode (EP1) critically depends on fibroblast growth factor receptor 1 (FGFR1) activity (Trokovic et al., 2005).

Using mouse embryos, the present study investigates potential contributions of FGF and BMP signals to the morphological establishment and/or to the neurogenesis of mammalian epibranchial placodes. Embryos were exposed to pan-FGFR or pan-bone morphogenetic protein receptor (BMPR) inhibitors in whole embryo culture experiments. Alternatively, anti-FGFR3 neutralizing antibodies were used to obtain more specific information on the relative contributions of distinct FGFRs. Treated embryos were evaluated histologically and statistically for the presence of Neurogenin (Ngn)2- and Ngn1-immunopositive epibranchial neuroblasts. It turned out that each of the three paired epibranchial placodes depends on different patterns of FGF and/or BMP signals, among other factors. At least in some cases, we were additionally able to distinguish (1) between the responses of Ngn2^+^ or Ngn1^+^ neuroblasts, (2) between the effects on epibranchial morphogenesis or neurogenesis, and (3) between the responses of epibranchial placodes and pharyngeal pouches, respectively.

## Materials and Methods

### Animals

Mated C57BL/6N mice were obtained from Janvier Labs (Le Genest-Saint-Isle, France). For embryo collection, these animals were killed 8.5–9 days post coitum by cervical dislocation. All handling steps were performed in accordance with animal welfare regulations and were approved by the responsible authority [Landesamt für Natur, Umwelt und Verbraucherschutz (LANUV), North Rhine-Westphalia, Germany; approval number: 84-02.05.50.16.013].

### Inhibitors and Antibodies

For blocking of FGF signaling, two different small molecule inhibitors were tested: SU5402 (3-[(3-(2-carboxyethyl)-4-methylpyrrol-2-yl)methylene]-2-indolinone; SML0443, Merck, Darmstadt, Germany) and PD173074 (1-*tert*-Butyl-3-[6-(3,5-dimethoxy-phenyl)-2-(4-diethylamino-butylamino)-pyrido[2,3-d]pyrimidin-7-yl]-urea; P2499, Merck). For use in whole embryo culture experiments, stock solutions of 100 mM SU5402 or 15 mM PD173074 were prepared in dimethyl sulfoxide (DMSO) (5179, Carl Roth, Karlsruhe, Germany). Both compounds were initially found to competitively block the adenosine triphosphate (ATP)-binding pocket of the tyrosine kinase domain of FGFR1 (Mohammadi et al., 1997, 1998). Later, it turned out that SU5402 and PD173074 additionally inhibit FGFR2, FGFR3, and FGFR4 (Grand et al., 2004; Koziczak et al., 2004; Trudel et al., 2004; St-Germain et al., 2009; Liu et al., 2013; Ranieri et al., 2016). In the context of our scientific questions, comparative testing of both pan-FGFR inhibitors is appropriate for at least two reasons. Firstly, SU5402 is the “classical” pan-FGFR inhibitor which has been applied in many different developmental studies (Raible and Brand, 2001; Corson et al., 2003) including those investigating neurogenesis in the cranial placodes of chick and zebrafish (for a review, see Lassiter et al., 2014). Secondly, PD173074, like SU5402, has already been used successfully in whole embryo cultures of mice. For example, addressing left-right axis formation, Oki et al. (2010) have demonstrated that PD173074 impairs gene expression patterns similar to *Fgf8* and *Fgfr1* knockouts.

For selective inhibition of the FGFR3 pathway, rat anti-FGFR3 neutralizing antibodies were deployed (MAB710, R&D Systems, Minneapolis, MN, United States, lot FTD0216021, RRID: AB_2103386). According to the manufacturer, these antibodies cross-react with the IIIb and IIIc isoforms of recombinant human and mouse FGFR3, and neutralize the bioactivity of mouse FGFR3. That, in fact, the anti-FGFR3 neutralizing antibodies used here actually block FGFR3 was also proven by Arnaud-Dabernat et al. (2007) taking the development and regeneration of mouse pancreatica as an example. Physiologically, activation of FGFR3 inhibits the expansion of immature pancreatic epithelia. Genetic silencing of FGFR3 in a mouse model of pancreas regeneration led to a 1.5-fold increase in the number of proliferating pancreatic ductal cells, as evidenced by BrdU incorporation. Correspondingly, when injected to adult mice for *in vivo* blockage of FGFR3, anti-FGFR3 neutralizing antibodies produced an approximate doubling of BrdU^+^ pancreatic epithelial cells. Arnaud-Dabernat et al. (2007) conclude that “FGFR3 attenuation by either genetic deletion or immune blockade led to a significant increase in epithelial cell expansion in pancreatic ducts.”

Given that only the first of three mouse epibranchial placodes is strongly dependent on FGFR1 activation (Trokovic et al., 2005), it was particularly important for us to apply anti-FGFR3 neutralizing antibodies that do not cross-react with FGFR1. This is exactly the requirement that anti-FGFR3 neutralizing antibodies obtained from R&D Systems fulfill (MAB710). Experimental evidence for this was provided by Shalhoub et al. (2011). These authors have studied FGF23+ membrane co-receptor alpha-Klotho signaling in osteoblastic MC3T3.E1 cells which express FGFR1, FGFR2, and FGFR3. It is demonstrated that the complete blockage of all FGFRs by the pan-FGFR inhibitor SU5402 (see above) causes a massive activation of bone-specific alkaline phosphatase. Comparable effects can be achieved neither by anti-FGFR2 neutralizing antibodies nor by anti-FGFR3 neutralizing antibodies. Consequently, the effect must be due to the activation of FGFR1, which remains undisturbed by the anti-FGFR3 neutralizing antibodies used here.

Inhibition of the BMP signaling pathway was performed with the small molecule inhibitor LDN193189 (4-[6-(4-(piperazin-1-yl)phenyl)pyrazolo[1,5-a]pyrimidin-3-yl] quinoline; SML0559, Merck) which was dissolved in water to produce a stock solution of 50 mM. LDN193189 is structurally derived from dorsomorphin that competitively blocks the ATP-binding pocket of the BMP type I receptor’s intracellular kinase domain (Chaikuad et al., 2012). Compared to dorsomorphin, LDN193189 demonstrates increased potency and pharmacokinetic stability (Cuny et al., 2008). In addition to its impact on BMP type I receptors [Activin receptor-like kinases (ALK) 1, 2, 3 and 6; Yu et al., 2008], LDN193189 efficiently binds to the BMP type II receptors Activin receptor IIA and IIB (Horbelt et al., 2015). LDN193189 has previously been applied to block BMP signaling during placode development in zebrafish embryos as well as during the formation and differentiation of human multipotent pre-placodal progenitors (Leung et al., 2013; Nikaido et al., 2017). To ensure that LDN193189 blocks the BMP pathway in cultured embryonic mice, we have tested whether this inhibitor is capable of preventing the expression of the BMP downstream effectors Msx1/2. The antibody used for this purpose (anti-Msx1/2 antibody 4G1, Developmental Studies Hybridoma Bank, Iowa City, IA, United States, lot 2/7/19, RRID: AB_531788) was raised against bacterially expressed chicken Msx2 and recognizes both Msx1 and Msx2 (Liem et al., 1995). Its specificity in chicken and mouse embryos has been further characterized in numerous publications, for example by comparison with *Msx1 in situ* hybridizations or Msx1-nlacZ expression patterns (Hu et al., 2008; Yamagishi et al., 2020). Physiologically, in E9.5 mouse embryos, Msx1/2 are expressed in dorsal parts of the hindbrain as well as in the mesenchyme of the first branchial arch (Coudert et al., 2005). These two expression sites are precisely what we can detect in our cultured control embryos by immunohistochemistry (Supplementary Figure 1). Silencing BMP4 by implantation of noggin-filled beads leads to a marked downregulation of *Msx1* in the mesenchyme of the first branchial arch (Tucker et al., 1998). Correspondingly, we demonstrate that the expression levels of Msx1/2 in branchial arch 1 decrease in a dose-dependent manner following exposure to increasing amounts of LDN193189 (Supplementary Figure 1). We conclude that LDN193189 indeed inhibits the BMP pathway.

For immunohistochemical detection of Ngn proteins, we applied either the goat anti-Ngn1 antibody (sc-19231, Santa Cruz, Dallas, TX, United States, lot C1215, RRID: AB_2298242) or the mouse anti-neurogenin-2 (Ngn2) antibody (clone 7G4, MAB3314, R&D Systems, lot WWI01, RRID: AB_2149520). The anti-Ngn1 antibody was raised against the peptide ARLQPLASTSGLSVPARRSAK mapping near the N-terminus of mouse Ngn1. It specifically detects a single band of about 19 kDa in Western blots of mouse brain extracts (manufacturer’s information). Specific labeling of Ngn1 in mouse tissue sections has already been demonstrated in our previous work on the development of lateral line placodes in mice (Washausen and Knabe, 2018). Additional evidence comes from studies on the regeneration of mouse olfactory epithelium following exposure to methyl bromide (Krolewski et al., 2013, and references therein). Here, findings demonstrated by anti-Ngn1 immunohistochemistry were compared to *Ngn1 in situ* hybridization data and, additionally, validated by studying the distribution patterns of enhanced green fluorescent protein (eGFP) in *Ngn1-eGFP* bacterial artificial chromosome transgenic mice. The anti-Ngn2 antibody was raised against a recombinant protein of the N-terminal basic helix-loop-helix domain of mouse *Ngn2* (Lo et al., 2002). It specifically detects Ngn2 in Western blots of embryonic mouse cortices (Ge et al., 2006), but does not produce immunolabeling in the retinae of postnatal *Ngn2* knock-out mice (Kowalchuk et al., 2018). Correspondingly, this antibody has been successfully used to characterize epibranchial neurogenesis in mice (Washausen and Knabe, 2013, 2018; Zhang et al., 2017). For specific labeling of neural crest cells, we used the mouse anti-Sox10 antibody sc-365692 from Santa Cruz Biotechnology (lot I0516, RRID: AB_10844002). All data required to characterize this antibody as well as the corresponding staining protocol have been provided in Washausen and Knabe (2018). The same applies to the anti-Pax8 antibody (clone BC12, ACI 438, Biocare Medical, Concord, CA, United States, lot 051712, RRID: AB_2864457) used to label epibranchial placode (precursor) cells (Washausen and Knabe, 2017).

### Whole Embryo Culture

Whole embryo culture was performed as has been described previously (Washausen and Knabe, 2018). The roller culture apparatus (BTC Engineering, Cambridge, United Kingdom) was connected to a gas mixing device (Gmix31, HiTec Zang, Herzogenrath, Germany) providing continuous gas supply (25 ml/min). Male Sprague Dawley rat serum was purchased from Janvier Labs and used as culture medium. Prior to the onset of embryo culture, heat-inactivated (56°C, 30 min) and centrifuged (2,000 × *g*, 10 min) culture medium was sterilely filtered, mixed with 0.25% antibiotic-antimycotic mix (15240096, Thermo Fisher Scientific, Schwerte, Germany), and equilibrated with 40% O_2_, 5% CO_2_, and 55% N_2_ for at least 1 h. Using a stereomicroscope (M165 FC, Leica, Wetzlar, Germany) in a laminar flow hood, mouse embryos were dissected in Hank’s balanced salt solution (L2035, Biochrom, Berlin, Germany) leaving the yolk sac and the ectoplacental cone intact. Embryos were then photographed with a digital camera (DFC450 C, Leica), and head lengths were measured using ImageJ (Rasband, 1997-2018). According to the developmental tables provided by van Maele-Fabry et al. (1993), all embryos were staged. Only those possessing 9–14 pairs of somites were transferred to the culture system (2–4 embryos per bottle, about 1 embryo/ml culture medium). Immediately prior to this transfer, culture bottles had been alternately supplemented either (1) with one of the two tested pan-FGFR inhibitors (SU5402, PD173074), or (2) with anti-FGFR3 neutralizing antibodies, or (3) with the pan-BMPR inhibitor LDN193189, or (4) exclusively with 0.1% DMSO for control. Working solutions (administered with 0.1% DMSO) were used as follows: 20, 40, or 80 μM SU5402; 0.5 or 2.5 μM PD173074; 5, 20, or 40 μg/ml anti-FGFR3 neutralizing antibodies; and 2, 5, or 10 μM LDN193189. Embryos were randomly assigned to each treatment group and incubated at 37.5°C (30 rpm) for 24 h in the dark. After 15 h, the continuous gas supply (40% O_2_, 5% CO_2_, and 55% N_2_) was modified to 70% O_2_ and 25% N_2_. At the end of the 24 h culture period, development of the embryos was analyzed according to the criteria published by van Maele-Fabry et al. (1990, 1993). Furthermore, stereomicrographs of the embryos were acquired to measure the yolk sac diameter as well as head and crown-rump lengths using ImageJ (Table 1). Following fixation in 4% paraformaldehyde in phosphate buffered saline at pH 7.4 for 24 h, the embryos were pre-embedded in 1% Seakem LE agarose (50001, Lonza, Köln, Germany) and, afterward, routinely embedded in Surgipath Formula “R” paraffin (3801450, Leica). Finally, whole specimens were serially sectioned at 5 μm. In order to facilitate staining of adjacent sections with different primary antibodies, consecutive serial sections were alternately placed on two sets of slides (Knabe et al., 2002).

**TABLE 1:** Developmental characteristics of 9–14 somite mouse embryos cultured for 24 h.

	Control	pan-FGFR inhibition					FGFR3 blocking			BMPR inhibition		
	DMSO	SU5402			PD173074		α-FGFR3			LDN193189		
	0.1% *n* = 14	20 μM *n* = 7	40 μM *n* = 16	80 μM *n* = 7	0.5 μM *n* = 16	2.5 μM *n* = 9	5 μg/ml *n* = 6	20 μg/ml *n* = 7	40 μg/ml *n* = 4	2 μM *n* = 5	5 μM *n* = 5	10 μM *n* = 8
Yolk sac diameter [mm]	3.2 3.0–3.3	3.1 3.0–3.1	3.0* 2.9–3.1	2.9* 2.8–3.0	3.0 2.9–3.3	2.7** 2.7–2.9	3.1 2.8–3.3	3.1 2.9–3.3	3.1 3.0–3.4	3.0 2.9–3.3	3.2 3.1–3.4	3.0 2.8–3.2
Crown-rump length [mm]	3.1 3.1–3.2	2.9* 2.7–3.0	2.7** 2.6–2.7	2.5** 2.3–2.7	3.0* 2.8–3.1	2.1** 2.1–2.2	3.1 2.8–3.6	3.0 2.7–3.4	3.1 3.0–3.3	3.3 2.8–3.5	3.2 2.9–3.4	2.9 2.9–3.3
Head length [mm]	1.9 1.8–1.9	1.6* 1.3–1.6	1.3** 1.2–1.3	1.1** 1.0–1.4	1.8 1.6–1.9	1.4** 1.4–1.4	1.7 1.5–2.0	1.7 1.4–1.9	1.8 1.6–2.0	1.9 1.5–2.0	1.7 1.6–1.9	1.6 1.5–1.8
Number of somites	27.3 26.0–28.0	24.5* 24.0–26.0	24.0** 23.0–26.0	22.0** 21.0–24.5	25.8 25.0–28.5	20.0** 19.5–21.0	26.5 25.5–29.0	26.5 24.5–27.0	28.5 26.5–29.5	26.0* 25.0–26.5	28.0 26.0–29.0	25.5* 24.0–26.3

Medians and interquartile ranges are given. Asterisks indicate significant differences between each treatment group and the respective control (Mann–Whitney test: *P < 0.05, **P < 0.001). BMPR, bone morphogenetic protein receptor; DMSO, dimethyl sulfoxide; FGFR, fibroblast growth factor receptor.

### Immunohistochemistry

Neurogenin-2 and Ngn1 immunostainings were carried out according to the protocols published in Washausen and Knabe (2013, 2018). In brief, for epitope retrieval, deparaffinized and rehydrated sections were treated in a high-pressure cooker in 10 mM citrate buffer (pH 6). Activity of endogenous peroxidases was blocked by incubation in 1% H_2_O_2_ and 0.3% Triton X-100 in Tris-buffered saline (TBS) (pH 7.4) for 30 min. Washing steps were carried out by rinsing slides three times with TBS for 5 min each. For Ngn2 immunohistochemistry, we used the mouse-on-mouse (M.O.M.) immunodetection kit (BMK-2002, Vector Laboratories, Burlingame, CA, United States) and performed protein blocking steps as well as incubations with anti-Ngn2 (dilution of 1:20,000, incubation overnight at 4°C) and secondary antibodies accordingly. The anti-Ngn1 antibody was applied at 1:100 in Dako REAL diluent (S202230-2, Agilent Technologies, Waldbronn, Germany) for 4 h at 37°C and detected with a biotinylated horse anti-goat antibody (1:100; BA-9500, Vector Laboratories, RRID: AB_2336123). Finally, for both Ngn2 and Ngn1 immunohistochemistry, sections were incubated with the avidin-biotin complex peroxidase reagent (PK-7100, Vector Laboratories) for 1 h. Following color reaction with 3,3′-diaminobenzidine (D5637, Merck), sections were counterstained in Mayer’s hematoxylin (Romeis, 1948), and embedded with DePeX mounting medium (18243, Serva, Heidelberg, Germany). Negative controls performed without primary antibodies revealed the absence of immunolabeling.

### Histological Analysis

In total, 104 completely serially sectioned embryos were examined. These embryos were distributed among the treatment groups as follows: pan-FGFR inhibitor SU5402 (20 μM: *n* = 7, 40 μM: *n* = 16, 80 μM: *n* = 7); pan-FGFR inhibitor PD173074 (0.5 μM: *n* = 16, 2.5 μM: *n* = 9); anti-FGFR3 neutralizing antibodies (5 μg/ml: *n* = 6, 20 μg/ml: *n* = 7, 40 μg/ml: *n* = 4); pan-BMPR inhibitor LDN193189 (2 μM: *n* = 5, 5 μM: *n* = 5, 10 μM: *n* = 8); control with 0.1% DMSO only (*n* = 14). Thickenings of the placodal ectoderm, outgrowth of the pharyngeal pouches and formation of the branchial membranes could be optimally diagnosed in the hematoxylin counterstained serial sections. Epibranchial neurogenic activity was assessed by separately analyzing Ngn2 and Ngn1 expression patterns on both sides of the embryonic body (section intervals: 10 μm). Only immunoreactive nuclei of those neuroblasts were counted that either resided in the epibranchial placode or were still in contact with the placode as emigrating ones. An exception to this is found in 3D reconstructions, where, due to the scientific question to be answered, an even stricter distinction must be made between (1) intraplacodal neuroblasts, (2) delaminating neuroblasts, and (3) neuroblasts that have already reached the mesenchyme adjacent to the placodes. In cases where exposure to the inhibitors suppressed the proper development of placodal thickenings and/or intact branchial membranes, prospective positions of the epibranchial placodes were determined by optically projecting the positions of underdeveloped pharyngeal pouches onto the opposing surface ectoderm. Furthermore, the entire branchial region was screened for Ngn2^+^ or Ngn1^+^ neuroblasts. Ngn2^+^ or Ngn1^+^ neuroblasts in the spinal cord as well as Ngn1^+^ neuroblasts in the trigeminal and otic placodes served as internal positive controls (Sommer et al., 1996; Fode et al., 1998; Ma et al., 1998). For each experimental group, 8–20 Ngn2- or Ngn1-immunostained epibranchial placodes were evaluated, respectively. Thus, the number of samples in all treatment groups corresponds to that which has been investigated in similar studies (e.g., Lassiter et al., 2009; Brown and Epstein, 2011).

### Statistics

Statistical analyses and creation of box plots were carried out in STATISTICA 13.3 (TIBCO Software, Munich, Germany). Initially, the Kolmogorov-Smirnov and the Levene’s test were applied to check normality and homogeneity of variances, respectively. Since not all of the cases examined satisfied both conditions, we selected the non-parametric Mann–Whitney test (two-sided) for comparison of the differences between general developmental characteristics or neuroblast numbers following different treatments. *P* values < 0.05 were regarded as statistically significant.

### Photomicrographs and Figures

Whole embryos were photographed using the Leica Application Suite (LAS) 4.6 software with a M165 FC stereomicroscope and a DFC450 C camera (both from Leica). Images of histological sections were acquired with the KS400 3.0 software using an Axioskop 2 MOT microscope and an AxioCam HR digital camera (Carl Zeiss, Göttingen, Germany). Following background and shading corrections in the respective imaging software, digital photographs were cropped and adjusted for brightness, color balance, and sharpness in Photo-Paint 2019 (Corel, Unterschleißheim, Germany). All image adjustments were carried out on the entire images without changing, removing or inserting specific features within the photographs. All figures and lettering were composed using CorelDraw 2019 (Corel). 3D reconstructions were created in the reconstruction and modeling software Free-D 1.15 (Andrey and Maurin, 2005).

## Results

**FIGURE 1:**
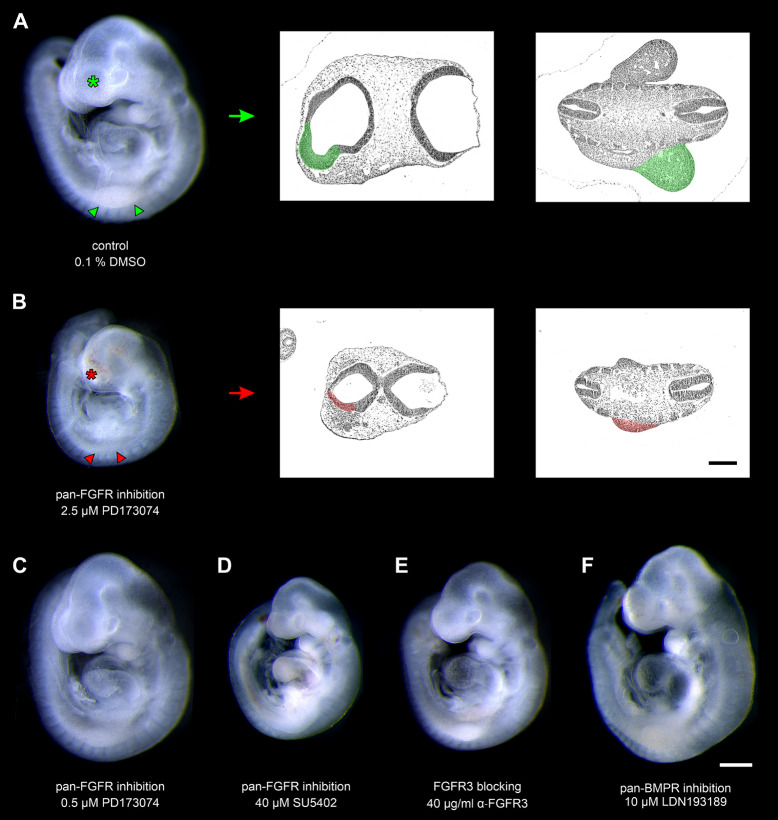
Nine to fourteen somite mouse embryos, cultured for 24 h. **(A)** Control embryo, exposed to the solvent dimethyl sulfoxide (DMSO) only, reveals age-appropriate telencephalic hemispheres (green asterisk) and upper limb buds (green arrowheads); for comparison (green arrow), see corresponding hematoxylin stained histological sections (green colored areas). **(B)** Treatment with 2.5 μM of the pan-FGFR inhibitor PD173074 considerably impairs the development of telencephalic hemispheres (red asterisk) and upper limb buds (red arrowheads); for comparison (red arrow), see corresponding hematoxylin stained histological sections (red colored areas). **(C)** Application of 0.5 μM PD173074 did not cause any obvious abnormalities. **(D)** Embryos incubated with 40 μM of the pan-FGFR inhibitor SU5402 exhibit general growth disturbances (also see Table 1). **(E,F)** Treatment with 40 μg/ml of anti-FGFR3 neutralizing antibodies or 10 μM of the pan-BMPR inhibitor LDN193189 were well tolerated. Scale bars: 500 μm for stereomicrographs **(A–F)**, 200 μm for light micrographs **(A,B)**.

Mouse embryos possessing 9–14 pairs of somites were cultured for 24 h in the presence of pan-FGFR inhibitors, anti-FGFR3 neutralizing antibodies, or the pan-BMPR inhibitor LDN193189, respectively (Figure 1 and Table 1). First, we have determined whether the pan-FGFR inhibitors SU5402 and PD173074 interfere with known FGF-dependent developmental steps of the forebrain and limb buds (Paek et al., 2009; Ornitz and Itoh, 2015; Figure 1A). Application of high doses of PD173074 (2.5 μM) disturbed the outgrowth of telencephalic hemispheres and limb buds (Figure 1B). In contrast, neither low dose exposure to PD173074 (0.5 μM; Figure 1C) nor application of SU5402 (20, 40, or 80 μM; Figure 1D) triggered such specific defects. Generalized growth retardation, as revealed by decreases in yolk sac diameter, crown-rump length, head length and number of somites (Table 1), was caused by SU5402 (Figure 1D) or high doses of PD173074 (2.5 μM; Figure 1B), but not by low doses of PD173074 (0.5 μM; Figure 1C) or by anti-FGFR3 neutralizing antibodies (Figure 1E). Embryos treated with the pan-BMPR inhibitor LDN193189 did not reveal statistically significant growth defects except for slight reductions of their somite numbers (Table 1 and Figure 1F).

**FIGURE 2:**
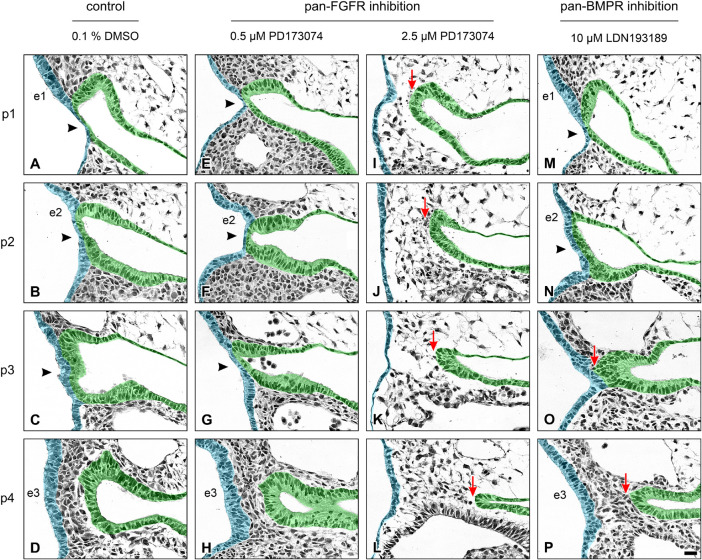
Impact of FGFR or BMPR inhibition on the development of pharyngeal pouches (p1–p4) in 9–14 somite mouse embryos, cultured for 24 h. Hematoxylin stained sections show the pharyngeal pouches (green), the overlying ectoderm (blue), the three epibranchial placodes (e1–e3), and the branchial membranes 1–3 (black arrowheads). **(A–D)** Control embryos: Age-appropriate lateral outgrowth of the pharyngeal pouches 1–4, whereby the latter physiologically does not fuse with the overlying ectoderm to form a “branchial membrane 4”. **(E–H)** Embryos incubated with 0.5 μM of the pan-FGFR inhibitor PD173074 develop largely normal pharyngeal pouches. **(I–L)** Treatment with 2.5 μM PD173074 prevents proper outgrowth of all pharyngeal pouches (red arrows). Neither branchial membranes nor epibranchial placodes are discernible. **(M–P)** Following application of the pan-BMPR inhibitor LDN193189, pharyngeal pouches 1 and 2 develop normally, whereas lateral outgrowth of the pharyngeal pouches 3 and 4 is impaired (red arrows) with pharyngeal pouch 3 contacting the ectoderm in one single histological serial section, if at all. Scale bar: 20 μm.

In a second step, we have investigated whether the formation of pharyngeal pouches is disturbed in the presence of pan-FGFR inhibitors, anti-FGFR3 neutralizing antibodies or the pan-BMPR inhibitor LDN193189. This question is justified for at least three reasons. Firstly, morphogenesis and neurogenesis of epibranchial placodes depend on signals produced by intact pharyngeal pouches (Ladher et al., 2010). Secondly, in zebrafish, FGF and BMP signaling promote pharyngeal pouch formation (Crump et al., 2004; Nechiporuk et al., 2005; Lovely et al., 2016; Li et al., 2019). Thirdly, pharyngeal pouches 3 and 4 are malformed in *Fgf8* hypomorphic mice (Abu-Issa et al., 2002; Frank et al., 2002). Control embryos demonstrated proper formation of all pharyngeal pouches (Figures 2A–D). Resembling *in utero* developed embryos, pharyngeal pouch 4 approached, but did not contact the branchial ectoderm (Figure 2D). Embryos exposed to low doses of PD173074 (0.5 μM) largely matched the controls according to morphological criteria (Figures 2E–H). As an exception, 3D reconstructions and additional serial section analyses revealed that 5 out of 20 body sides (25%) of the embryos treated with low doses of PD173074 presented varying degrees of (mostly discrete) segmentation defects of the pharyngeal pouches 2 and 3 (see below). In only 2 other body sides (10%) these defects were severe enough to no longer allow a full distinction between the branchial membranes 2 and 3. In contrast, high doses of PD173074 (2.5 μM) impaired the lateral outgrowth of all four pharyngeal pouches (Figures 2I–L). Consequently, branchial membranes were absent. Morphologically normal pharyngeal pouches were again found in all embryos incubated with SU5402 or anti-FGFR3 neutralizing antibodies, respectively (data not shown). Finally, embryos exposed to moderate or high, but not low, doses of the pan-BMPR inhibitor LDN193189 (5 or 10 μM) displayed obvious defects of the pharyngeal pouches 3 and 4, but not 1 and 2 (Figures 2M–P).

**FIGURE 3:**
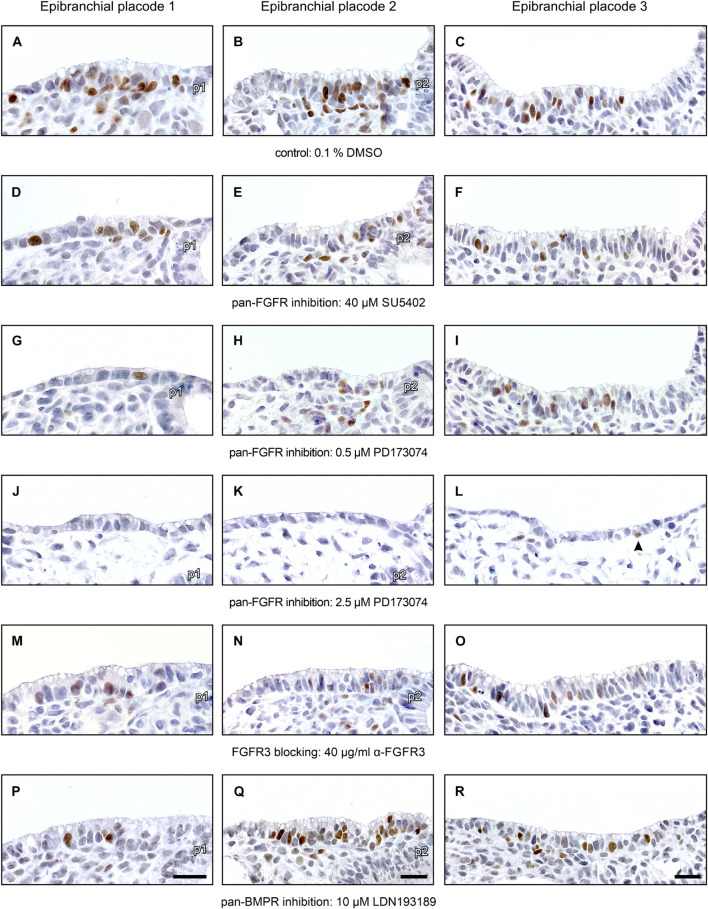
Impact of FGFR and BMPR inhibition on epibranchial placode morphogenesis and neurogenesis in 9–14 somite mouse embryos, cultured for 24 h. Anti-Neurogenin-2 (Ngn2) immunohistochemistry (brown precipitate); shown are maximum numbers of Ngn2^+^ neuroblasts found per section. **(A–C)** Control placodes reveal high-grade thickened pseudostratified epithelium (2–4 cell nuclei) with 12–16 Ngn2^+^ neuroblasts. **(D–F)** Pan-FGFR inhibitor SU5402 (40 μM) causes moderate reductions in placode thickness and statistically significant decreases in neuroblast numbers in the epibranchial placodes 1 and 2, but not 3 (also see Figure 4). **(G–I)** Pan-FGFR inhibitor PD173074 (0.5 μM) elicits strong decreases in the thickness of the epibranchial placodes 1 and 2 as well as statistically significant decreases in neuroblast numbers in all three epibranchial placodes (also see Figure 4). **(J–L)** High doses of PD173074 (2.5 μM) result in the complete absence of high-grade thickened epibranchial placodes. Only single Ngn2^+^ neuroblasts can be detected, if at all (arrowhead in panel **L**). **(M–O)** Anti-FGFR3 neutralizing antibodies (40 μg/ml) reduce Ngn2^+^ neuroblast numbers, but do not have negative effects on placode thickness. **(P–R)** Following pan-BMPR inhibition (10 μM LDN193189), epibranchial placode 1 reveals reduced numbers of Ngn2^+^ neuroblasts, but normal morphology **(P)**; epibranchial placode 2 lacks any obvious impairment **(Q)**; epibranchial placode 3 simultaneously presents slight reductions in Ngn2^+^ neuroblast numbers and placode thickness **(R)**. p1, p2, pharyngeal pouches 1 and 2. Scale bars: 20 μm.

Next, we have analyzed whether pan-FGFR inhibitors, anti-FGFR3 neutralizing antibodies or the pan-BMPR inhibitor LDN193189 affect epibranchial placode morphogenesis. Resembling *in utero* developed E9.5 to E10 embryos (Washausen and Knabe, 2013, 2017), control embryos cultured for 24 h revealed three pairs of high-grade thickened epibranchial placodes (pseudostratified epithelium with up to four rows of nuclei, Figures 3A–C). In contrast, treatment with 40 μM SU5402 already causes slight decreases in the thickness of EP1 and EP2 (Figures 3D–F). Correspondingly, embryos treated with low doses of PD173074 (0.5 μM) presented considerably thinned-out EP1 and EP2 (Figures 3G,H), whereas EP3 remained high-grade thickened (Figure 3I). High doses of PD173074 (2.5 μM) prevented the development of high-grade ectodermal thickenings in the positions of all three epibranchial placodes (Figures 3J–L). Embryos incubated with anti-FGFR3 neutralizing antibodies exhibited three regularly formed epibranchial placodes (Figures 3M–O). BMPR inhibition with LDN193189 (10 μM) led to an obvious, but moderate thinning only in EP3 (Figures 3P–R).

**FIGURE 4:**
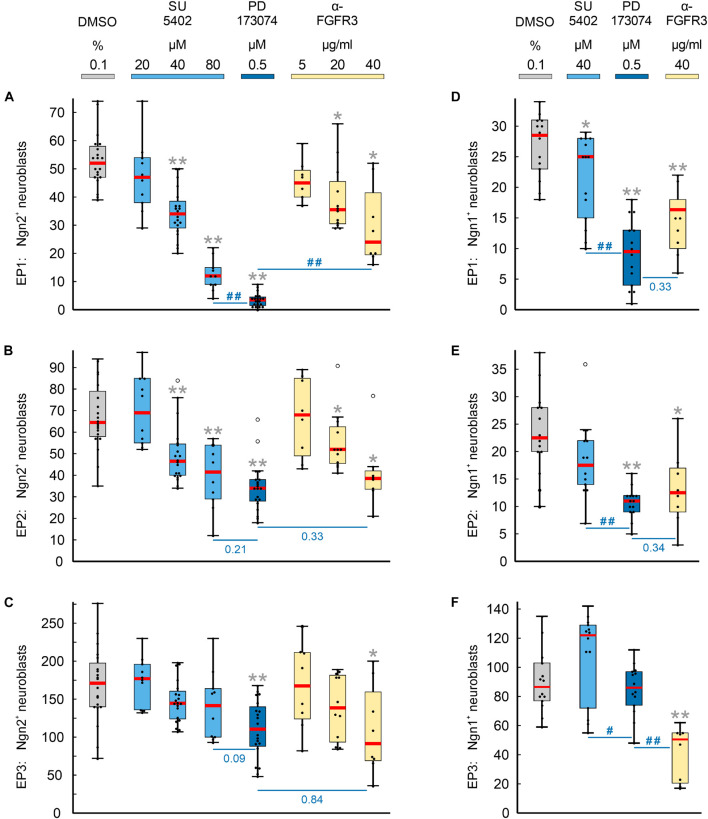
Impact of FGFR inhibition on the neurogenesis of epibranchial placodes (EP) in 9–14 somite mouse embryos, cultured for 24 h. Box plots indicate the numbers of Neurogenin (Ngn)2^+^ **(A–C)** or Ngn1^+^ **(D–F)** neuroblasts in EP1 **(A,D)**, EP2 **(B,E)**, or EP3 **(C,F)** following exposure to either dimethyl sulfoxide (DMSO) only (control; gray), or increasing doses of pan-FGFR inhibitor SU5402 (light blue), or 0.5 μM of pan-FGFR inhibitor PD173074 (dark blue), or increasing concentrations of anti-FGFR3 neutralizing antibodies (yellow). Significant differences between each treatment group and the controls are indicated by gray asterisks (*P < 0.05, **P < 0.001; Mann–Whitney test). Additionally, PD173074-treated embryos were compared to groups exposed to either highest levels of SU5402 or anti-FGFR3 antibodies (significant differences: #*P* < 0.05, ##*P* < 0.001; or specific P values, respectively). Whiskers, lower and upper extremes; box limits, 25th and 75th percentiles; red center lines, medians; black dots, data points; open circles, outliers.

Numbers and distribution patterns of Ngn2^+^ epibranchial neuroblasts in control embryos (Figures 3A–C, 4A–C) matched with those found in *in utero* developed embryos (Washausen and Knabe, 2013). Application of increasing doses of SU5402 resulted in graded decreases of Ngn2^+^ neuroblasts in all three epibranchial placodes (Figures 3D–F, 4A–C). Taking the respective medians as reference values (Table 2), decreases were strongest in EP1 (−77%), much more moderate in EP2 (−36%), and without statistical significance in EP3 (−18%). That, in fact, EP1 is much more dependent on FGF signaling than EP2 and EP3 came out even clearer when embryos were exposed to low doses of PD173074 (Figures 3G–I, 4A–C). Compared to controls, decreases of Ngn2^+^ neuroblasts amounted to −93% (EP1), −47% (EP2), and −35% (EP3), respectively. Compared to the highest dose of SU5402 used here, exposure to low doses of PD173074 led to significantly stronger reduced numbers of Ngn2^+^ neuroblasts only in EP1.

**TABLE 2:** Relative changes of neuroblast numbers in the epibranchial placodes of 9–14 somite mouse embryos cultured for 24 h.

	Control	pan-FGFR inhibition				FGFR3 blocking		BMPR inhibition	
	DMSO	SU5402		PD173074		α-FGFR3		LDN193189	
Ngn2^+^	0.1% *n* = 20	40 μM *n* = 20	80 μM *n* = 10	0.5 μM *n* = 20	2.5 μM *n* = 10	20 μg/ml *n* = 12	40 μg/ml *n* = 8	5 μg/ml *n* = 10	10 μg/ml *n* = 16
EP1	**52**	−34.6% **	−76.9% **	−93.2% **	−100% **	−31.7% *	−53.8% *	−84.6% **	−90.4% **
EP2	**64.5**	−27.9% **	−35.7% **	−47.3% **	−100% **	−19.4% *	−40.3% *	−6.2%	−13.2%
EP3	**171**	−15.5%	−17.5%	−35.4% **	−98.8% **	−19.0%	−46.5% *	−35.4% *	−39.2% **

**Ngn1^+^**	**0.1%**	**40 μM**		**0.5 μM**	**2.5 μM**	**40 μg/ml**		**10 μg/ml**	
	***n* = 14**	***n* = 14**		***n* = 14**	***n* = 8**	***n* = 8**		***n* = 10**	

EP1	**28.5**	−14.0% *		−66.7% **	−57.9% **	−50.9% **		−86.0% **	
EP2	**22.5**	−22.2%		−51.1% **	−100% **	−44.4% *		−26.6%	
EP3	**86.5**	+41.0%		−0.6%	−96.5% **	−41.6% **		−44.5% **	

**FIGURE 5:**
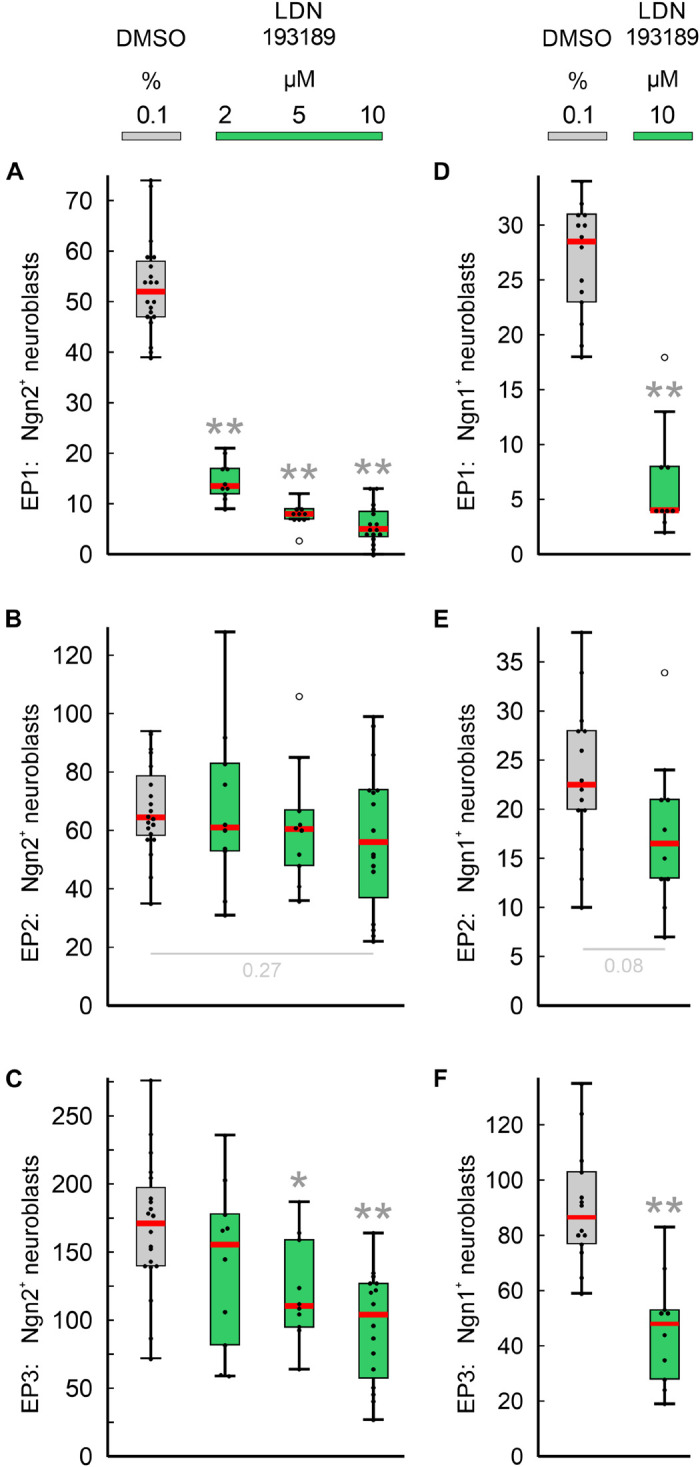
Impact of pan-BMPR inhibition on the neurogenesis of epibranchial placodes (EP) in 9–14 somite mouse embryos, cultured for 24 h. Box plots indicate the numbers of Neurogenin (Ngn)2^+^ **(A–C)** or Ngn1^+^ **(D–F)** neuroblasts in EP1 **(A,D)**, EP2 **(B,E)**, or EP3 **(C,F)** following exposure to either dimethyl sulfoxide (DMSO) only (control; gray), or increasing doses of LDN193189 (green). Significant differences between each treatment group and the controls are indicated by gray asterisks (*P < 0.05, **P < 0.001; Mann–Whitney test). Whiskers, lower and upper extremes; box limits, 25th and 75th percentiles; red center lines, medians; black dots, data points; open circles, outliers.

Medians (bold numbers, provided for controls) served as reference values for calculating the changes (%) in neuroblast numbers for each treatment group. Asterisks indicate significant differences between each treatment group and the respective control (Mann–Whitney test: *P < 0.05, **P < 0.001; also see Figures 4, 5). BMPR, bone morphogenetic protein receptor; DMSO, dimethyl sulfoxide; FGFR, fibroblast growth factor receptor; Ngn1, Neurogenin1; Ngn2, Neurogenin2.

Whether and to what extent subtypes of FGFRs are expressed in the epibranchial placodes of mice is largely unknown. As an exception, Trokovic et al. (2005) demonstrated that neurogenesis in EP1, but not EP2 and EP3, substantially depends on FGFR1. We have investigated the impact of FGFR3 blockage on epibranchial neurogenesis. Exposure to high doses of anti-FGFR3 neutralizing antibodies (40 μg/ml) significantly decreased the number of Ngn2^+^ neuroblasts in all three epibranchial placodes (Figures 3M–O, 4A–C and Table 2). However, only in EP1 did these decreases lag significantly behind those achieved by low doses of PD173074 (Figure 4A). We conclude that FGF-dependent neurogenesis in EP2 and EP3 predominantly occurs via FGFR3 whereas, in EP1, it is controlled by (at least) FGFR1 (Trokovic et al., 2005) and FGFR3 (present results).

In the epibranchial neuroblasts of mice, expression of *Ngn1* is downstream of *Ngn2* in EP1, EP2 and, partly, in EP3. In the latter, a second subpopulation of neuroblasts upregulates *Ngn1* independently of *Ngn2* (Fode et al., 1998). We aimed to find out, whether incubation with pan-FGFR inhibitors, anti-FGFR3 neutralizing antibodies or the pan-BMPR inhibitor LDN193189 provides additional evidence for the existence of differently regulated subpopulations of epibranchial neuroblasts. In line with findings obtained from *in utero* developed embryos (Fode et al., 1998), the number of Ngn1^+^ neuroblasts in our control embryos reached only about 55% (EP1), 35% (EP2), and 52% (EP3) of the respective numbers of Ngn2^+^ neuroblasts (Figure 4 and Table 2). SU5402 (40 μM) caused significant decreases of Ngn1^+^ neuroblasts only in EP1 (Figures 4D–F). Exposure to low doses of PD173074 significantly reduced the number of Ngn1^+^ neuroblasts in EP1 and EP2 (Figures 4D,E). It was only EP3 that deviated from this “pattern” in that neither SU5402 (40 μM) nor low doses of PD173074 resulted in significant decreases of Ngn1^+^ neuroblasts (Figure 4F and Table 2). Exposure to anti-FGFR3 neutralizing antibodies (40 μg/ml) significantly reduced the number of Ngn1^+^ (and Ngn2^+^) neuroblasts in all three epibranchial placodes (Figures 3M–O, 4). However, when comparing the effects resulting from treatments with low doses of PD173074 or anti-FGFR3 neutralizing antibodies, respectively, we found placode-specific responses of Ngn1^+^ neuroblasts. In EP1 and EP2, statistically significant decreases in Ngn1^+^ neuroblasts were not observed (Figures 4D,E). In contrast, significantly stronger reductions in the number of Ngn1^+^ neuroblasts occurred in EP3 following incubation with anti-FGFR3 neutralizing antibodies (40 μg/ml; Figure 4F).

Next, we have investigated whether the pan-BMPR inhibitor LDN193189 affects epibranchial neurogenesis in mice (Figure 5). Significant decreases in Ngn2^+^ neuroblasts were already observed following treatments with 2 μM (EP1, Figure 5A) or 5 μM (EP1, EP3; Figures 5B,C). Correspondingly, incubation with 10 μM LDN193189 significantly reduced the numbers of Ngn2^+^ and Ngn1^+^ neuroblasts in EP1 (approximately −90%) and EP3 (approximately −40%; Table 2). In contrast, LDN193189 was unable to significantly lower the numbers of Ngn1^+^ and Ngn2^+^ neuroblasts in EP2 (Figures 5B,E and Table 2).

Finally, we would like to point out that in almost all cases where FGFR inhibitors led to massive disturbances in the outgrowth of the pharyngeal pouches, Ngn2^+^ or Ngn1^+^ neuroblasts were virtually absent from the expected positions of the three epibranchial placodes (Figures 3J–L, and data not shown). As an exception, approximately 40% of Ngn1^+^ neuroblasts persisted in EP1 (Table 2, also see discussion).

## Discussion

**FIGURE 6:**
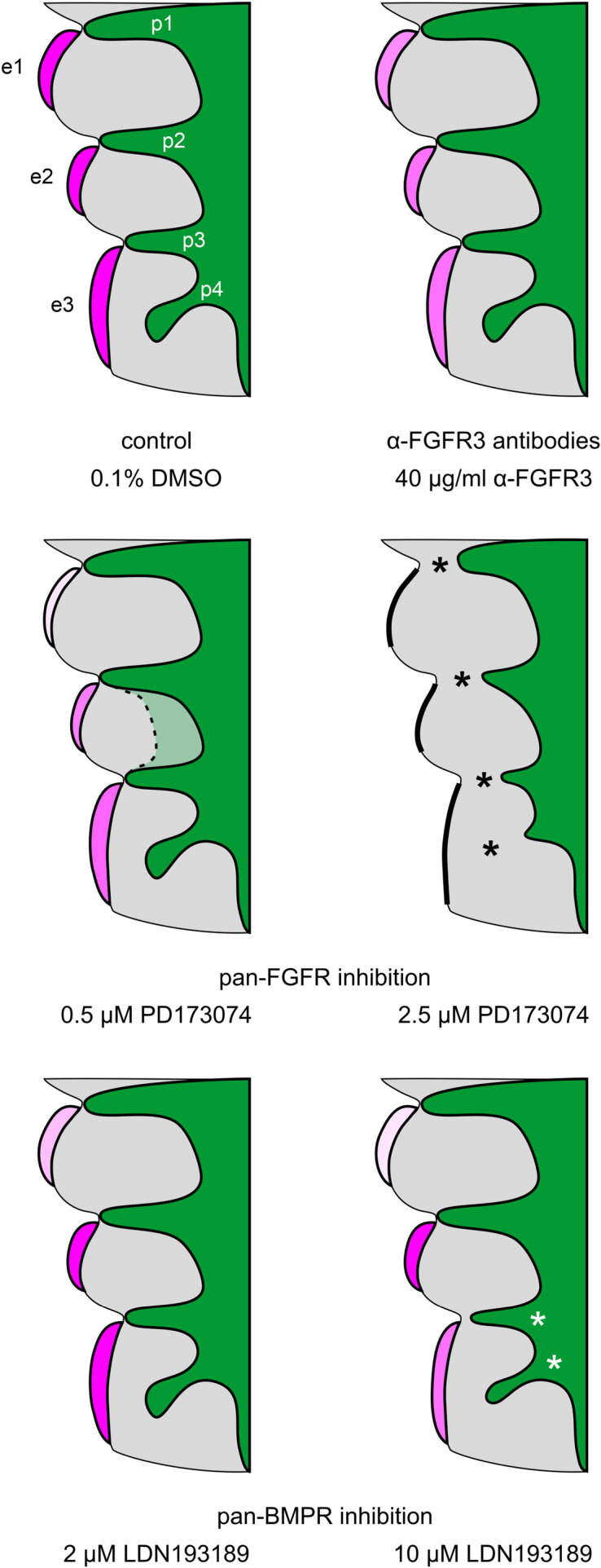
Summary scheme: Impact of FGFR and BMPR inhibition on epibranchial placode morphogenesis and neurogenesis in 9–14 somite mouse embryos, cultured for 24 h. e1, e2, e3, epibranchial placodes 1, 2, and 3; p1, p2, p3, p4, pharyngeal pouches 1, 2, 3, and 4; green, endoderm; gray, branchial arches. Increasing intensities of purple color indicate increasing numbers of Neurogenin2^+^ epibranchial neuroblasts. Different degrees of placode thickness are represented schematically. Thick black lines, complete absence of epibranchial placodes; black or white asterisks, impaired lateral outgrowth of the pharyngeal pouches. The faint green area enclosed by the dashed line indicates that pharyngeal pouches 2 and 3 may show (mostly discrete) segmentation defects in some of the embryos treated with 0.5 μM of the pan-FGFR inhibitor PD173074 (for details, see text).

The present work shows that, in mice, epibranchial placode development is dependent on both FGF and BMP signaling as revealed by whole embryo culture experiments using pan-FGFR inhibitors (SU5402, PD173074), anti-FGFR3 neutralizing antibodies and the pan-BMPR inhibitor LDN193189 (Figure 6). The most parsimonious hypothesis would have been that all three epibranchial placodes would respond in the same way to identical treatments. However, this hypothesis turned out to be unfounded. Furthermore, depending on the respective treatment, impaired epibranchial placodes occurred either in the presence or absence of malformed pharyngeal pouches which act as epibranchial signaling centers (Ladher et al., 2010). We therefore discuss potential implications of FGF and BMP signaling for epibranchial placode development in five consecutive steps.

### Methodological Considerations

The pan-FGFR inhibitors SU5402 and PD173074 both block the ATP-binding pocket of the tyrosine kinase domains of the four known FGFR subtypes (Mohammadi et al., 1997, 1998; Raible and Brand, 2001; Kyono et al., 2011). SU5402 has often been used in studies dealing with placode development (Lassiter et al., 2014). In the meantime, it has become clear that substantial limitations must be considered when using SU5402. Firstly, SU5402 not only interferes with FGFRs but also potently blocks several off-target kinases. Secondly, SU5402 is rather toxic in the range of effective doses (Gudernova et al., 2016). Correspondingly, we observed growth impairments of our embryos already at 40 μM SU5402 (Figure 1D and Table 1), a dose that is commonly applied during the developmental period studied here (Corson et al., 2003; Calmont et al., 2006; Oki et al., 2010; Wang et al., 2015). Thirdly, SU5402 either works or remains without effect in a context-dependent manner (Oki et al., 2010). Fourthly, SU5402 suffers from poor tissue penetration (Ahrens and Schlosser, 2005). Consistent with these limitations, we were unable to reproduce FGF-dependent malformations of the forebrain (Paek et al., 2009), limb buds (Ornitz and Itoh, 2015) and pharyngeal pouches (Abu-Issa et al., 2002; Frank et al., 2002) using SU5402. However, compared to PD173074 which is less toxic and approximately 1000-fold more potent (Pardo et al., 2009; Lamont et al., 2011), SU5402 dose-dependently produced identical, albeit attenuated effects in the epibranchial placodes of mice (present results). Another argument in favor of the alternative use of SU5402 is that only through the separate use of SU5402 and PD173074 we were able to discover that some epibranchial placodes depend completely on FGF signals, while others do so only partially. Thus, low doses of PD173074 completely suppressed the production of Ngn2^+^ neuroblasts in EP1 but, in EP2 and EP3, did not statistically significantly enhance the moderate decrease of Ngn2^+^ neuroblasts achieved by the highest doses of SU5402 used here (80 μM).

Another methodological consideration addresses the question whether pharmacological inhibition of the FGF signaling pathway specifically affects branchial mesoderm patterning. The underlying hypothesis is based on the fact that *Fgf10* is upregulated in the branchial mesoderm by *Fgf3* and *Fgf8* (Alvarez et al., 2003; Wright and Mansour, 2003; Wright et al., 2004; Ladher et al., 2005; Aggarwal et al., 2006). FGF signaling in turn causes an upregulation of *Msx1* in the branchial mesoderm during exactly the developmental period we are looking for (Wakamatsu et al., 2019; one embryonic day later: Chen et al., 1996). Exposure of our embryos to 0.5 μM of the pan-FGFR inhibitor PD173074 already leads to a discrete attenuation of the Msx1/2 signal (Supplementary Figure 2). Correspondingly, only a few weak Msx^+^ mesoderm cells remain in the (hypoplastic) branchial arches after treatment with 2.5 μM PD173074 (Supplementary Figure 2). These findings suggest that PD173074 indeed specifically interferes with FGF signaling in the branchial arch mesoderm of cultured mouse embryos. Quite similar dose-dependent malformations caused by PD173074 will be discussed in the context of pharyngeal pouch formation.

### Impact of FGF Signaling on Epibranchial Placode Development

We first discuss to what extent low doses of PD173074 impair epibranchial placode development in mice. This treatment resulted in a statistically significant decrease of neuroblasts in EP1 (Ngn2^+^, Ngn1^+^), EP2 (Ngn2^+^, Ngn1^+^), and EP3 (Ngn2^+^). Overall, the numbers of Ngn2^+^ neuroblasts declined along a rostrocaudal gradient (EP1: −93%, EP2: −47%, and EP3: −35%; Table 2). Individually different responses of the epibranchial placodes to pan-FGFR inhibition were also found in zebrafish when SU5402 was applied in an equivalent time window (onset of treatment: 19–24 hpf). Thus, FGF-dependent neuroblast production is absent in EP2 and EP3_1_, but decreases only mildly in EP1 as well as in the common anlage of EP3_2_ to EP3_4_ (Nechiporuk et al., 2005). Nevertheless, interspecies differences also may exist. For it was only in mice that reductions in neuroblast production were accompanied by significant or even complete reductions in placode thickness (EP1, EP2: Figures 3G,H; also see EP1 to EP3 following high doses of PD173074: Figures 3J–L; EP1, EP2 following SU5402: Figures 3D,E). Instead, structurally unaltered epibranchial placodes were observed in SU5402-treated zebrafish (Nechiporuk et al., 2005). One possible explanation for this latter difference might be that proliferation and neurogenesis follow different time schedules in the epibranchial placodes of zebrafish and mice. Correspondingly, in zebrafish, an earlier onset of SU5402 exposure (10–16.5 hpf) completely suppresses both epibranchial morphogenesis and neurogenesis (Nechiporuk et al., 2007). Conversely, a late onset (26 hpf) does not affect the epibranchial placodes in any way (Nechiporuk et al., 2005).

We further demonstrate that, in mice, FGFR3 is involved to varying degrees in the neurogenesis of distinct epibranchial placodes. Thus, in EP2 and EP3, both treatment with anti-FGFR3 neutralizing antibodies and treatment with low doses of PD173074 reduced the number of Ngn2^+^ neuroblasts by about the same amount. We conclude that FGFR3 almost completely mediates FGF-dependent effects on Ngn2^+^ neuroblasts in these two placodes. In contrast, only about one third of the FGF-dependent production of Ngn2^+^ neuroblasts requires the involvement of FGFR3 in EP1. Our results perfectly complement earlier findings on the roles of FGFR1 in mice. Here, FGFR1 deficiency impairs Ngn2 expression in EP1, but not in EP2 and EP3. Consequently, production of Ngn2^+^ neuroblasts in EP1 must critically depend on the combined action of at least FGFR1 (Trokovic et al., 2005) and FGFR3 (present results). In this context, interspecies differences once again become apparent. Namely, during epibranchial neurogenesis in zebrafish, FGF-dependent signals are exclusively transmitted via FGFR1 (Nechiporuk et al., 2005).

In the mice studied here, FGFR3 dependence of Ngn2^+^ and Ngn1^+^ epibranchial neuroblasts, respectively, turned out to be different in degree. FGF-dependent effects on Ngn2^+^ neuroblasts are either fully (EP2, EP3) or only to about one third (EP1) mediated via FGFR3. In FGF-dependent Ngn1^+^ neuroblasts, however, a practically completely FGFR3-mediated signal transmission occurs in EP1 and EP2. EP3 is somehow out of line as far as the Ngn1^+^ neuroblasts are concerned. On the one hand, treatment with anti-FGFR3 neutralizing antibodies, as in EP1 and EP2, causes significant declines in the number of Ngn1^+^ neuroblasts. On the other hand, significant decreases of Ngn1^+^ neuroblasts observed in EP1 and EP2 under the influence of pan-FGFR inhibitors did not manifest in EP3. This supposedly paradoxical situation might be explained by the hypothesis that, in EP3, FGFRs other than FGFR3 physiologically block the production of Ngn1^+^ neuroblasts. Consequently, blockage of these other FGFRs by pan-FGFR inhibitors would increase the number of Ngn1^+^ neuroblasts and, thus, would compensate for any losses caused by the simultaneous blockage of FGFR3. That different FGFRs indeed exert opposing effects on specific cell populations is known from other contexts (Feng et al., 2015). For example, epithelial-mesenchymal transitions in various tumors are promoted by FGFR1 and FGFR4, but are suppressed by FGFR2. Whether, in EP3, “paradoxically” regulated Ngn1^+^ neuroblasts (present results) belong to the subpopulation of Ngn2-independent Ngn1^+^ neuroblasts discovered by Fode et al. (1998) remains to be determined. In this context, we will also make a more refined attempt to distinguish between subpopulations of neuroblasts expressing Ngn1, Ngn2, or both.

Wright and Mansour (2003) have examined the roles of *Fgf3* and *Fgf10* in ear development. Unlike our approach, this study worked with knockout mice. This means that potentially disturbing influences on the development of epibranchial placodes which, however, were not explicitly addressed by Wright and Mansour (2003), already affect the induction of epibranchial placodes, but not primarily their neurogenesis. Analysis of the published images shows that *Pax2*^+^ epibranchial placodes are neither detectable in *Fgf3*^+/–^, *Fgf10*^–/–^ mice nor in *Fgf3*^–/–^, *Fgf10*^+/–^ mice. These results are in principle consistent with our current findings. This also applies to the study conducted by Freter et al. (2008). Here, knockdown constructs were used to demonstrate that, in chicken embryos, induction and further development of the epibranchial placodes can be completely disrupted by reducing mesodermal *Ffg3* and *Fgf19* expression. However, given that, again, FGF signaling had been switched off at a much earlier developmental period compared to our approach, neither of the two studies can determine, whether and which components of the FGF signaling pathway are responsible for neurogenesis in individual epibranchial placodes at a later stage.

**FIGURE 7:**
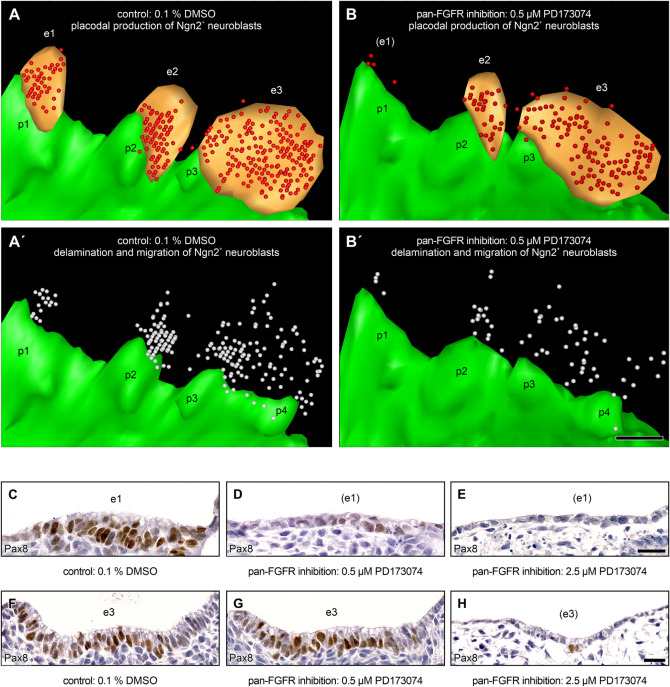
Production and delamination of epibranchial Ngn2^+^ neuroblasts in 9–14 somite mouse embryos, cultured for 24 h. 3D reconstructions show that, compared to DMSO control embryos **(A)**, the number of Ngn2^+^ neuroblasts (red spheres) in the epibranchial placodes 1, 2, and 3 (e1, e2, e3; orange) decreases to varying degrees (e1 > > e2 > e3) after exposure to 0.5 μM of the pan-FGFR inhibitor PD173074 (**B**, also note loss of ectodermal thickening in the position of e1; for details, see Figure 4 and Table 2). **(A’,B’)** Largely proportionally to the number of Ngn2^+^ intraplacodal neuroblasts, the number of Ngn2^+^ delaminating neuroblasts respectively of those that have already been deposited within the mesenchyme adjacent to the epibranchial placodes (both populations shown jointly as white spheres) also decreases. **(C–H)** Furthermore, treatment with PD173074 reduces, again roughly proportionally to the number of Ngn2^+^ intraplacodal neuroblasts, the number of Pax8^+^ intraplacodal (precursor) cells in a dose-dependent manner. p1, p2, p3, and p4, pharyngeal pouches 1, 2, 3, and 4 (green). Scale bars: 100 μm **(A–B’)**, 20 μm **(C–E,F–H)**.

Finally, we pursue the question whether FGF signaling might be of particular importance for the delamination of epibranchial neuroblasts. Indeed, Lassiter et al. (2009) observed that *Fgfr4* is maximally expressed in the ophthalmic trigeminal placode of chicken embryos during the delamination period. In addition, these authors were able to prove, both by genetic silencing of *Fgfr4* and by pharmacological inhibition of FGFR4 using the pan-FGFR inhibitor SU5402, that FGF signals are responsible for neuroblast delamination as well as for neuroblast differentiation. However, the experimental design did not allow to decide whether or not both processes depend on each other. To address the points raised by Lassiter et al. (2009), we generated 3D reconstructions of mouse control embryos as well as of embryos treated with 0.5 μM of the pan-FGFR inhibitor PD173074. In the control embryos, high numbers of Ngn2^+^ neuroblasts were found in all three epibranchial placodes as well as in the approximate future positions of the associated geniculate, petrosal, and nodose ganglia, respectively (Figures 7A,A’). Exposure to 0.5 μM PD173074 leads to strong (EP1) or only moderate (EP2 > EP3) decreases in the number of Ngn2^+^ placodal neuroblasts (Figures 3G–I, 4A–C, 6, 7B, and Table 2). Correspondingly, extremely low (developing geniculate ganglion) or still moderate numbers of Ngn2^+^ neuroblasts (immature petrosal and nodose ganglia) were observed in the underlying mesenchyme (Figure 7B’). The situation is therefore formally similar to the one described by Lassiter et al. (2009). In the ophthalmic trigeminal placode, however, the number of placodal cells, as revealed by the early marker Pax3, remained constant under the influence of FGF inhibition, while it decreased in the underlying mesenchyme. This can be regarded as a clear indication of a delamination disorder. It must be added, however, that an isolated delamination disorder is out of question here, as this should have been accompanied by an increase in the number of Pax3^+^ premigratory neuroblasts. In mouse epibranchial placodes, the case is certainly different. Here, the number of premigratory placodal cells, as revealed by the early marker Pax8, strongly decreases following FGF inhibition (Figures 7C–H, and data not shown). Consequently, FGF inhibition already negatively affects the number of placodal progenitor cells, which ultimately reduces neuroblast delamination, albeit for other reasons.

### Contributions of BMP Signaling to Epibranchial Placode Development

In zebrafish and chicken embryos, blockage of BMP signaling completely eliminates neurogenesis in all epibranchial placodes (Holzschuh et al., 2005; Kriebitz et al., 2009). At variance with this pattern, exposure of embryonic mice to the pan-BMPR inhibitor LDN193189 caused different degrees of decrease in the numbers of Ngn2^+^ neuroblasts in each individual epibranchial placode: EP1 (−90%), EP2 (statistically insignificant), EP3 (−39%). Thus, neurogenesis of Ngn2^+^ neuroblasts in EP1 and EP3 performed largely as we had observed under the influence of low doses of the pan-FGFR inhibitor PD173074 (EP1: −93%, EP3: −35%). In contrast, neurogenesis of Ngn2^+^ neuroblasts in EP2 is strongly supported by FGF signals, but practically independent of BMP signals. The latter statement is substantiated by two pieces of evidence. Firstly, the BMPR inhibitor LDN193189 blocks kinase activity of all known BMP type 1 receptors as well as of two out of three known BMP type II receptors (Mohedas et al., 2013; Horbelt et al., 2015). Secondly, the only BMP type II receptor that cannot be blocked by LDN193189 (BMPRII) is not expressed in the developing epibranchial placodes of mice (Figures 4–6 in Danesh et al., 2009).

Compared to Ngn2^+^ neuroblasts, Ngn1^+^ neuroblasts responded almost identically to LDN193189 (Figure 5); the resulting decreases amounted to −86% (EP1), −45% (EP3), or are statistically insignificant (EP2). Thus, resembling Ngn2^+^ neuroblasts, Ngn1^+^ neuroblasts are either dependent on both BMP and FGF signaling (EP1, EP3) or to about 50% on FGF signals, in the absence of effective BMP signals (EP2). We therefore assume that neurogenesis in EP2 is additionally regulated by at least a third signaling pathway.

So far, we have considered possible influences of FGF and/or BMP signaling on epibranchial neurogenesis. FGF signaling, possibly via receptors other than FGFR3, additionally appears to support the development and/or maintenance of the placodal thickenings (Figures 3, 6). In contrast, BMP signals hardly participate in the structural assembly of EP1 and EP2, even at concentrations that cause a virtually complete extinction of neurogenesis in EP1. As an exception, EP3 is somewhat reduced in thickness under the influence of LDN193189, but this BMP-dependent effect will be discussed in the context of concomitant damages of the pharyngeal pouches. Whether FGF and BMP signals reinforce or override each other during epibranchial placode development, as they do in other contexts (Hayashi et al., 2001; Maier et al., 2010), will be addressed in future studies.

### Critical Appraisal of the Developmental Profiles of Individual Epibranchial Placodes

**FIGURE 8:**
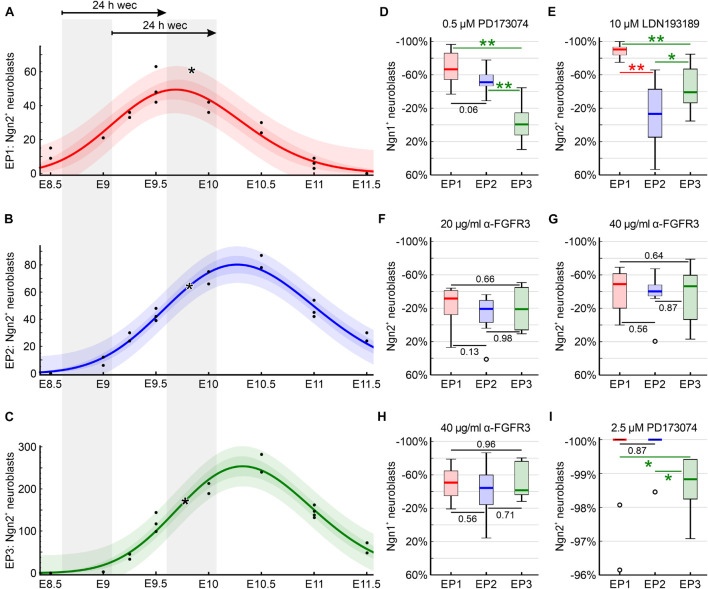
Developmental profiles of the epibranchial placodes of C57BL/6N mice (embryonic days (E) 8.5 to E11.5) as reflected by earlier reconstructions of Ngn2^+^ intraplacodal neuroblasts (Washausen and Knabe, 2013) recounted for present purposes (*n* = 11 mouse embryos with 22 body sides). **(A–C)** Using CurveExpert Professional (Hyams Development, Chattanooga, TN, United States), regression curves were generated that represent the developmental profiles of the epibranchial placodes 1, 2, and 3 (EP1, red; EP2, blue; EP3, green). Confidence bands (medium red, blue, or green) most likely contain 95% of all values (black dots). Prediction bands (faint red, blue, or green) indicate the range in which 95% of all future values will fall. Here, embryos possessing 9–14 pairs of somites were included in our whole embryo cultures (WECs). This range is indicated by the left of the two gray columns and is correlated with the embryonic age plotted on the *x*-axis. The right gray column marks the period within which embryos were removed from the culture after 24 h. Asterisks indicate the respective medians of Ngn2^+^ neuroblasts for EP1, EP2, and EP3 of DMSO control embryos (Table 2). **(D–I)** Box plots display the relative changes in the numbers of Ngn2^+^ **(E–G,I)** or Ngn1^+^ **(D,H)** neuroblasts following exposure to either the pan-FGFR inhibitor PD173074 **(D,I)**, to anti-FGFR3 neutralizing antibodies **(F–H)**, or to the pan-BMPR inhibitor LDN193189 **(E)**, respectively (see Table 2, also for *n* values). Significant differences between EP1 (red), EP2 (blue), and/or EP3 (green) are indicated by asterisks (*P < 0.05, **P < 0.001; Mann–Whitney test). Whiskers, lower and upper extremes; box limits, 25th and 75th percentiles; red, blue, or green center lines, medians; open circles, outliers. **(D,E)** Plausibility checks demonstrate that EP2 and EP3, which almost synchronously produce neuroblasts, by no means always react in the same way to certain inhibitors. **(D,F–I)** Conversely, EP1, which slightly precedes EP2 and EP3 in terms of neuroblast production, does not always deviate in its behavior toward certain inhibitors from EP2 and/or EP3 (for details, see text).

Epibranchial placodes form with a rostral to caudal sequence from EP1 to EP3. Consequently, it needs to be checked whether our postulate that BMP and FGF signaling pathways play at least partially different roles in each of the three epibranchial placodes may unintentionally reflect different developmental profiles, or in other words, rostrocaudal sensitivity differences of EP1, EP2, and EP3. To this end, we referred back to one of our previous papers (Washausen and Knabe, 2013) and graphically documented the developmental profile of each epibranchial placode (Figures 8A–C). Our diagrams demonstrate that EP1, EP2, and EP3 start generating Ngn2^+^ neuroblasts almost simultaneously between E8.5 and E9. The rostrocaudal developmental gradient becomes slightly more evident when neuroblast production reaches maximum numbers at about E9.7 (EP1) or E10.3 (EP2, EP3). Exposure of all three epibranchial placodes to the various inhibitors or neutralizing antibodies occurred uniformly immediately after the onset of neuroblast production. At this time, all three epibranchial placodes were equally well accessible to our reagents, since both overgrowth and/or invagination of the caudal epibranchial placodes begin only after the end of culture. Another consensus between EP1, EP2, and EP3 is that substantial periods of exposure to inhibitors or neutralizing antibodies coincided with the steep increase in neuroblast production. The only relevant difference is that it was solely EP1 that was cultivated beyond its production maximum. However, this difference is put into perspective by the fact that, in the two caudal epibranchial placodes, end of culture occurred at latest when 96.6% (EP2) or 91% (EP3) of the production maximum had been reached. Furthermore, the developmental profiles of EP2 and EP3 overlap almost completely. Critically appraising all these facts, we do not believe that the chosen culture period causes any systematic bias.

This view is supported by several plausibility arguments. Thus, EP2 and EP3, which show virtually identical developmental profiles (Figures 8B,C), should behave most similarly to each other in case that all epibranchial placodes would share identical dependencies on the FGF and/or BMP signaling pathways. However, EP2 and EP3 behave significantly differently (1) with respect to the reduction of Ngn2^+^ neuroblasts following exposure to 10 μM of the pan-BMPR inhibitor LDN193189 and (2) with respect to the reduction of Ngn1^+^ neuroblasts after treatment with 0.5 μM of the pan-FGFR inhibitor PD173074 (Figures 8D,E). A second testable hypothesis is that EP1 generally behaves differently from EP2 and/or EP3 because of its (slightly) different developmental profile. However, completely against this hypothesis stands the fact that the response behavior of EP1 does not differ significantly from EP2 and/or EP3 when placodes are treated (1) with 20 and/or 40 μg/ml neutralizing anti-FGFR3 antibodies (Ngn2, Ngn1; EP2, EP3), (2) with 2.5 μM PD173074 (Ngn2; EP2), or (3) with 0.5 μM PD173074 (Ngn1; EP2) (Figures 8D,F–I). In summary, we find no evidence arising from the plausibility checks that our results are picking up a rostrocaudal difference in epibranchial placode development and that our inhibition experiments may reflect a rostrocaudal difference in sensitivity that underlies the differences seen.

### Impact of FGF and BMP Signaling on Pharyngeal Pouch Development

**FIGURE 9:**
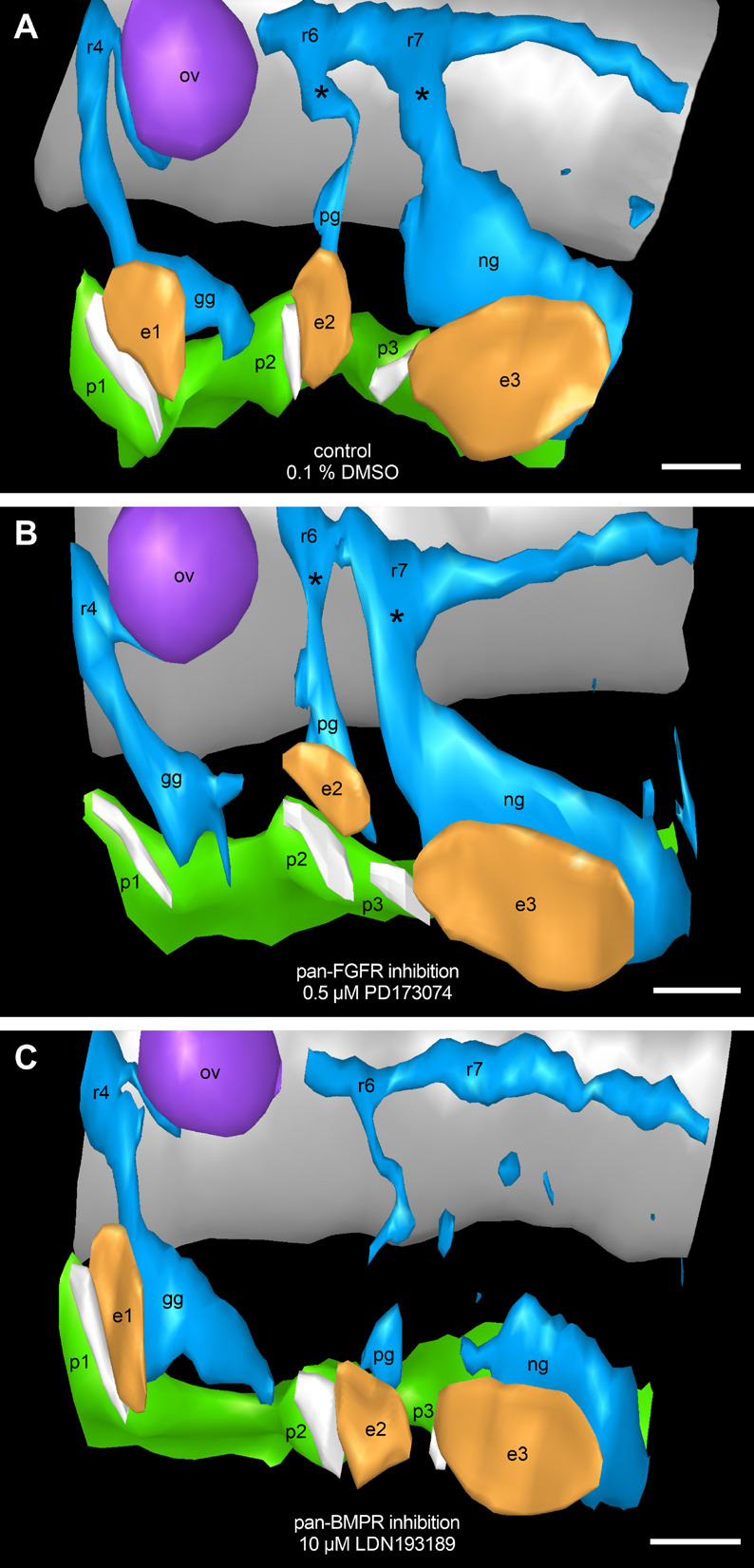
Assessment of potential FGF and/or BMP dependencies of neural crest streams emerging segmentally from the rhombomeres 4, 6, and 7. 9–14 somite mouse embryos, cultured for 24 h. Our 3D reconstructions show that neural crest streams (blue) emerge from rhombomeres 4, 6, and 7 (r4, r6, and r7), and give rise to the proximal ganglia (asterisks) of the cranial nerves IX and X as well as to the geniculate, petrosal, and nodose ganglia (gg, pg, and ng) both in DMSO control embryos **(A)** and after exposure to 0.5 μM of the pan-FGFR inhibitor PD173074 **(B)**. Treatment with 10 μM of the pan-BMPR inhibitor LDN193189, like genetic silencing of the BMP pathway (Kanzler et al., 2000), leads to the partial loss of neural crest streams and proximal ganglia originating from r6 and, to a greater extent, r7 **(C)**. In at least some of the PD173074 treated embryos (for details, see text), pharyngeal pouches 2 and 3 as well as branchial membranes 2 and 3 have approached each other beyond the normal level. Also note loss of ectodermal thickening in the position of epibranchial placode 1. e1, e2, e3, epibranchial placodes 1, 2, and 3 (orange); ov, otic vesicle (purple); p1, p2, p3, pharyngeal pouches 1, 2, and 3 (green); white stripes adjacent to the epibranchial placodes, branchial membranes 1, 2, and 3; gray, hindbrain. Scale bars: 100 μm.

Pharyngeal pouches are indispensable signaling centers for the development of epibranchial placodes (Holzschuh et al., 2005; Kriebitz et al., 2009; Ladher et al., 2010). In embryonic mice, incubation with low doses of the pan-FGFR inhibitor PD173074 did not result in generalized malformations of these signaling centers. It must be added, however, that 5 out of 20 body sides (25%) showed mild to moderate and 2 out of 20 body sides (10%) even severe segmentation defects of the pharyngeal pouches 2 and 3 (Figures 6, 7B, 9B). All in all, we therefore assume that impaired epibranchial placodes primarily arose from FGFR blockage in the placodal ectoderm. However, we cannot exclude that hitherto undetected subtle changes in the contact zone between the pharyngeal endoderm and the branchial ectoderm also contributed to the developmental anomalies. Indeed, using zebrafish *van gogh* (*Tbx1*) mutants, Holzschuh et al. (2005) discovered that even slight increases or decreases of this contact area lead to significant increases or decreases of epibranchial neurogenesis, respectively.

When high doses of PD173074 were applied to cultured mouse embryos, lateral outgrowth of all four pharyngeal pouches was impeded. Furthermore, the degree of placode damage by far exceeded that caused by low doses of PD173074. We hypothesize that, in these severe cases, complete suppression of epibranchial placode development cannot be ascribed to the blocking of ectodermal FGFRs alone. Instead, any pouch-derived signal should become less effective due to the increased distance to its ectodermal targets. Correspondingly, FGF ligands maximally bridge distances of about 16 cell diameters in zebrafish (Scholpp and Brand, 2004) and, in *Xenopus laevis*, BMP signals cannot be transmitted further than approximately 5 to 10 cell diameters (Dosch et al., 1997).

In embryonic mice, pharyngeal pouch formation either was largely undisturbed or strongly impaired depending on the dose of pan-FGFR inhibitor PD173074 administered. Similarly, development of the pharyngeal pouches remained unaffected following exposure to low doses of the pan-BMPR inhibitor LDN193189 (2 μM), but became increasingly disturbed upon incubation with progressively higher doses (5 μM, 10 μM). However, while in the case of high doses of PD173074 all four pharyngeal pouches were affected, high doses of LDN193189 impaired the outgrowth of the pharyngeal pouches 3 and 4, but left the pharyngeal pouches 1 and 2 unaffected. Correspondingly, in zebrafish, application of the BMPR inhibitor dorsomorphin elicits a stronger disturbance of the pharyngeal pouches 3–6 when compared to the pharyngeal pouches 1 and 2 (Lovely et al., 2016). We cannot presently decide whether these differential outcomes are dose-dependent, or whether different sets of molecular signals contribute to the formation of distinct pharyngeal pouches.

Malformations of the pharyngeal pouches may be caused either by the disturbance of direct endodermal effects of BMP and/or FGF signaling, or indirectly by abnormalities that primarily affect the formation of neural crest cells and/or their segment-specific migration into the branchial arches (but see Veitch et al., 1999). To test the latter hypothesis, routinely counterstained serial sections of all control embryos as well as of all mouse embryos treated with either 0.5 μM of the pan-FGFR inhibitor PD173074 or with 2, 5, or 10 μM of the pan-BMPR inhibitor LDN193189 were analyzed. In addition, serial sections of three embryos (control, 0.5 μM PD173074 and 10 μM LDN193189) were studied immunohistochemically using antibodies against Sox10 and 3D reconstructed (Figure 9). Neither control embryos nor embryos treated with PD173074 showed major deviations from the typical neural crest patterning (Figures 9A,B). In contrast, about 90% of the embryos exposed to 10 μM LDN193189 presented massive proximally accentuated defects of the glossopharyngeal and vagal neural crest streams (Figure 9C). Furthermore, basically identical defects (data not shown) occurred at 2 μM LDN193189 (50%) and roughly reached the percentage found at 10 μM LDN193189 already at 5 μM LDN193189 (80%).

The first “gain” of these analyses is that they additionally validate the basic experimental approach of this work. In fact, application of LDN193189 in our whole embryo cultures triggers exactly those segment-specific neural crest cell defects that are caused by genetic silencing of the BMP signaling pathway (Kanzler et al., 2000). Secondly, our 3D reconstructions of LDN193189-treated embryos demonstrate that glossopharyngeal and vagal neural crest defects coincide with a discretely reduced approach of the pharyngeal pouches 3 and 4 to the opposing branchial ectoderm. However, we can rule out in all probability that mis-migrated neural crest cells form a cellular barrier that may prevent fusion between pharyngeal endoderm and branchial ectoderm. Consequently, with the current state of knowledge, we favor a scenario in which the observed malformations of the pharyngeal pouches result from the pharmacological blockage of endodermal BMP receptors.

Our findings support the assumption that the pharyngeal pouch signaling center contributes essentially to epibranchial placode neurogenesis (Begbie et al., 1999; for a review, see Ladher et al., 2010). Indeed, massive disruption of pharyngeal pouch formation by exposing mouse embryos to the pan-FGFR inhibitor PD173074 resulted in massive deficiencies of all three epibranchial placodes (Figure 6). In line with this observation, less severe damage to the pharyngeal pouches 3 and 4 caused by the pan-BMPR inhibitor LDN193189 led to moderate impairments of EP3 (see above). However, there is also evidence that other signaling centers may be involved in epibranchial neurogenesis in addition to the pharyngeal pouches. Thus, in zebrafish *casanova* (*Sox32*) mutants, disruption of the pharyngeal pouches results on the one hand in complete (EP2, EP3_1_), on the other hand in only moderate decreases in the numbers of neuroblasts (EP1, EP3_2__–__4_; Holzschuh et al., 2005; Nechiporuk et al., 2005). Furthermore, McCarroll and Nechiporuk (2013) were able to demonstrate that, in zebrafish, progenitor cells of both the otic and the anterior lateral line placodes serve as epibranchial signaling centers. Whether anlagen of lateral line placodes that we discovered in mice (Washausen and Knabe, 2018) also may execute such signaling functions will be investigated in subsequent studies. However, there is a second possible explanation for the “atypical” findings detected in EP1 and EP3_2__–__4_ of zebrafish *casanova* mutants. Indeed, different pharyngeal pouches could employ different sets of short- and long-range signals to regulate epibranchial neurogenesis (Schlosser, 2003; Kriebitz et al., 2009), the effectiveness of which would be limited to different degrees in cases of impaired pharyngeal pouch formation.

## Data Availability Statement

The raw data supporting the conclusions of this article will be made available by the authors, without undue reservation.

## Ethics Statement

The animal study was reviewed and approved by Landesamt für Natur, Umwelt und Verbraucherschutz (LANUV), North Rhine-Westphalia, Germany; approval number: 84-02.05.50.16.013.

## Author Contributions

Both authors listed have made a substantial, direct and intellectual contribution to the work, and approved it for publication.

## Conflict of Interest

The authors declare that the research was conducted in the absence of any commercial or financial relationships that could be construed as a potential conflict of interest.

## Publisher’s Note

All claims expressed in this article are solely those of the authors and do not necessarily represent those of their affiliated organizations, or those of the publisher, the editors and the reviewers. Any product that may be evaluated in this article, or claim that may be made by its manufacturer, is not guaranteed or endorsed by the publisher.
