# Supplementary material for: Responses of Epibranchial Placodes to Disruptions of the FGF and BMP Signaling Pathways in Embryonic Mice
Source: Front Cell Dev Biol. 2021 Sep 13;9:712522. doi: 10.3389/fcell.2021.712522 (PMC8473811; doi:10.3389/fcell.2021.712522)
Supplement: Supplementary file 1 [file Data_Sheet_1.PDF]

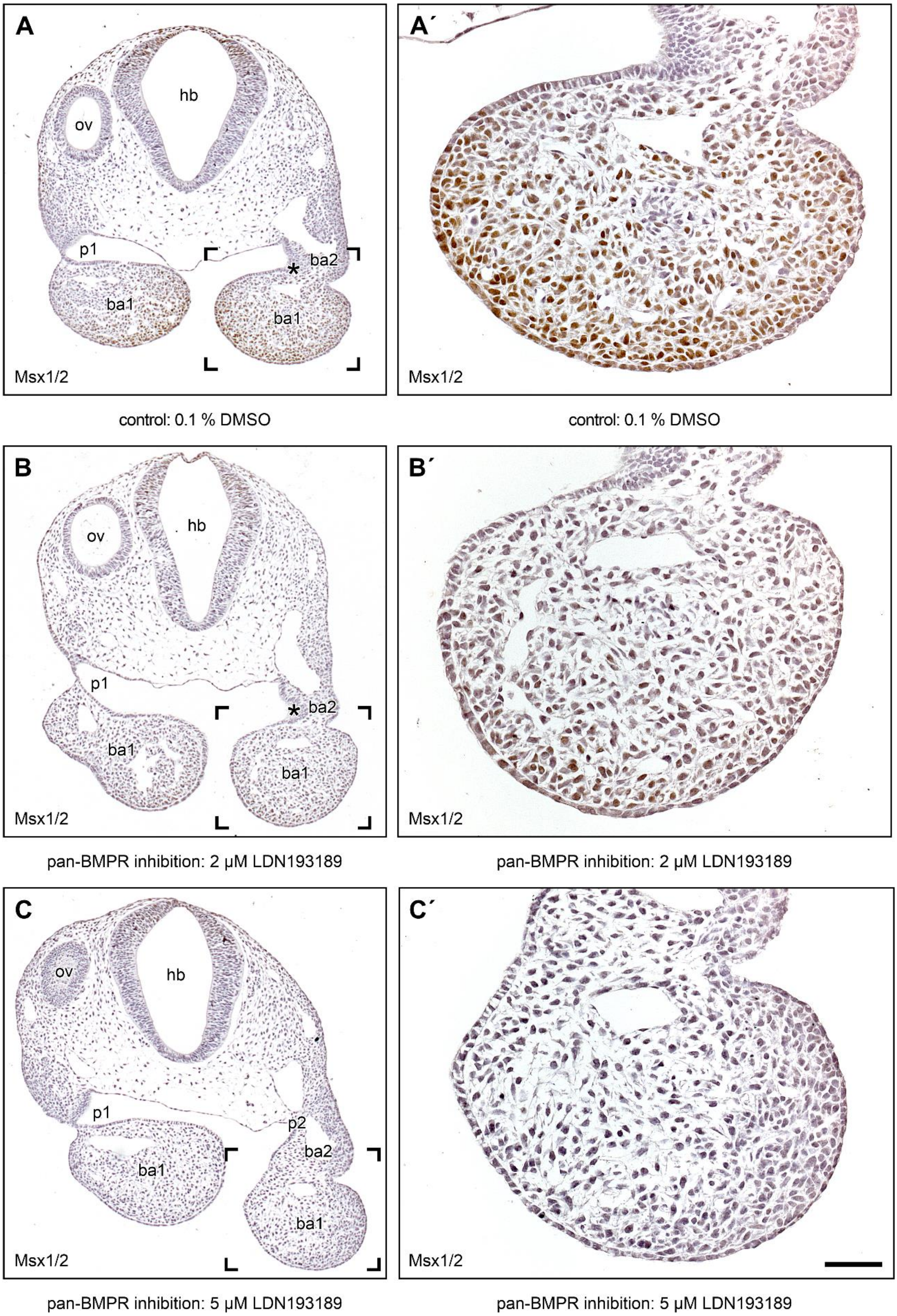

Supplementary Figure 1

Supplementary Figure 1. BMP-dependent patterning of branchial arch mesenchyme by its downstream effector Msx1/2 in 9-14 somite mouse embryos, cultured for 24 hours. Msx1/2-immunolabelled and hematoxylin stained sections show that Msx1/2 is strongly expressed in the dorsal hindbrain (hb) and in the branchial arch 1 (ba1) of DMSO control embryos (**A**, inset enlarged in **A´**), and dose-dependently decreases following exposure to 2 µM (**B**, inset enlarged in **B´**) or 5 µM of the pan-BMPR inhibitor LDN193189 (**C**, inset enlarged in **C´**). Asterisks, tangentially sectioned pharyngeal pouch 1; ba2, branchial arch 2; ov, otic vesicle; p1, p2, pharyngeal pouches 1 and 2. Scale bar: 50 µm.

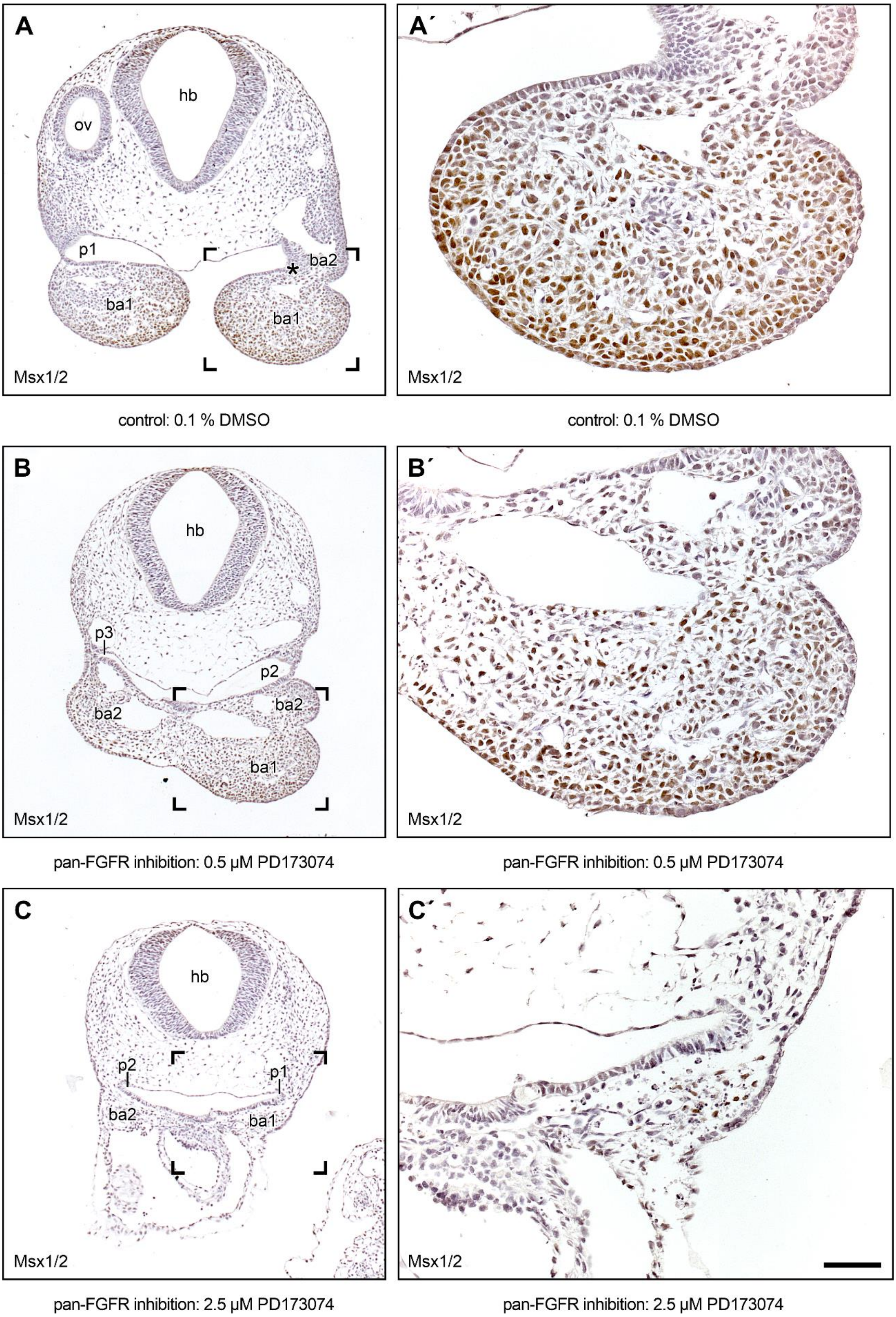

Supplementary Figure 2

Supplementary Figure 2. FGF-dependent patterning of branchial arch mesenchyme by its downstream effector Msx1/2 in 9-14 somite mouse embryos, cultured for 24 hours. Msx1/2-immunolabelled and hematoxylin stained sections show that Msx1/2 is strongly expressed in the dorsal hindbrain (hb) and in the branchial arch 1 (ba1) of DMSO control embryos (**A**, inset enlarged in **A´**), and dose-dependently decreases following exposure to 0.5 µM (**B**, inset enlarged in **B´**) or 2.5 µM of the pan-FGFR inhibitor PD173074 (**C**, inset enlarged in **C´**). Downregulation of Msx1/2 is associated with marked hypoplasia of the branchial arches. Asterisk, tangentially sectioned pharyngeal pouch 1; ba2, branchial arch 2; ov, otic vesicle; p1, p2, p3, pharyngeal pouches 1, 2, and 3. Scale bar: 50 µm.
